# Determinants of prebiotic vegetable consumption: the extended theory of planned behaviour

**DOI:** 10.1186/s13690-020-00408-z

**Published:** 2020-05-13

**Authors:** V. J. V. Broers, S. Van den Broucke, O. Luminet

**Affiliations:** 1 Psychological Sciences Research Institute, UCLouvain, 10 Place Cardinal Mercier, 1348 Louvain-la-Neuve, Belgium; 2 Fund for Scientific Research (FRS-FNRS), Brussels, Belgium

**Keywords:** Prebiotic, Vegetables, Determinants, TPB, Representative

## Abstract

**Background:**

Prebiotic vegetables such as leek and salsify may contribute to preventing obesity by changing the composition of the gut microbiota. To increase consumption of prebiotic vegetables, the aim of the study was to document the prevalence and determinants of (prebiotic) vegetable consumption.

**Methods:**

An online, correlational questionnaire was administered to participants using a mixed approach (1078 online, 200 face-to-face). Participants were a representative sample (gender, age, level of education, province, population density and (un)employment) of 1278 adults of the Walloon region in Belgium. The frequency and determinants of prebiotic vegetable consumption were measured using an extension of the Theory of Planned Behaviour including habits, actual control and compensatory health beliefs. Descriptive analyses were performed followed by hierarchic multiple regression analyses.

**Results:**

The descriptive results showed that for all categories (leek, salsify, vegetables in general) an improvement in both intentions and prevalence of the actual behaviour is necessary to experience the health benefits of (prebiotic) vegetables. Intentions and habits were important predictors of consumption for all types of vegetables, and hedonic attitudes and subjective norms were important predictors of intention. Perceived control and rational attitudes were predictors of intention to consume only for vegetables in general. Finally, environmental factors such as price, availability and actual control predict consumption but their influence differs depending on the vegetable.

**Conclusions:**

The findings can be used to inform interventions that aim to increase (prebiotic) vegetable consumption. Umbrella terms such as ‘healthy food’ or ‘vegetables’ do not capture the differences between the specific foods regarding the demographic and socio-psychological determinants of their consumption. This is the first research to investigate the determinants of prebiotic vegetable consumption.

## Background

Obesity is one of the biggest challenges for health worldwide. Between 1975 and 2016, worldwide obesity has nearly tripled to over 650 million adults suffering from this condition [1]. This increase can also be noticed in Belgium, leading to 15.9% in the adult population suffering from obesity in 2018 [2]. To prevent this chronic disease, it is recommended by the World Health Organization to be physically active, reduce the intake of saturated fat, sugar and salt, and to ensure a minimum intake of 400 g of fruits and vegetables per day [3]. Overweight is not only caused by an unhealthy diet however: the composition and/or activity of the microbiota in the gut is also related to overweight [4]. Influencing the gut microbiota can therefore also be a potential avenue for the prevention and decrease of obesity.

One way to change the composition of the gut microbiota is to enhance the consumption of prebiotics as part of the diet [4, 5]. Prebiotics are non-digestible food ingredients that stimulate the growth and/or activity of bacterial species that are already present in the gut of the host [6]. Prebiotics are not to be confused with probiotics, which are live microbial food supplements [7]. Inulin-type fructans (ITF) are certain types of fibres that are known for their prebiotic capacities: they can modulate gut microbiota, energy metabolism and improve glucose metabolism [8]. These ITF are typically found in “prebiotic” vegetables such as leek, Jerusalem artichoke, artichoke, and salsify [9]. Increased consumption of these prebiotic vegetables could therefore help to decrease overweight and obesity.

To enhance the consumption of these vegetables, it is recommendable to make use of psychological models of preventive behaviour change. These models typically focus on the socio-cognitive factors that determine intentional behaviour such as food choice. Among the most prominent socio-cognitive models to study health-related behaviour change are the Trans-Theoretical Model of change (TTM) [10] and the Theory of Planned Behaviour (TPB) [11]. The Trans-Theoretical Model of change is a so-called “stage model” that identifies five stages in the process of behaviour change: pre-contemplation, contemplation, preparation, action and maintenance. The Theory of Planned Behaviour, on the other hand, is an explanatory model, which identifies behavioural intention as a proxy of behaviour, and posits that intention in turn is influenced by belief-based attitudes, subjective norms and perceived control [11]. One systematic review on the psychosocial determinants of fruit and vegetable intake in an adult population concluded that the TPB was one of the most preferred social cognitive theories to predict behaviour and intention [12]. Another systematic review and meta-analysis [13] that investigated the association between TPB variables and discrete food choice behaviours corroborates this finding by reporting medium to large effect sizes of associations between TPB variables with both intention and behaviour. The association between intention and behaviour was *r* + = 0.45. Attitudes had the strongest association with intention (*r* + = 0.54) followed by perceived behavioural control (PBC; *r* + = 0.42) and subjective norms (*r* + = 0.37). The association between PBC and behaviour was *r* + = 0.27. Other variables have been suggested in addition to the TPB to increase the explained variance for food behaviour. For instance, specific beliefs called compensatory health beliefs (CHB) have been proposed to explain why people sometimes make conscious justifications about unhealthy food choices by referring to the idea that certain unhealthy but pleasurable behaviours can be compensated for by engaging in healthy behaviours [14]. The performance of most behaviours also depends to some extent on non-motivational factors however, such as actual control [11]. Furthermore, repeated behaviour is expected to result in a habit*,* which refers to repeated behaviour occurring in stable contexts [15] of which the person is unaware and that is difficult to control. Habits are closely related to past behaviour, but aim to explain past experience that leads to habitual rather than reasoned responses [11]. Given the consistent support for the predictive validity of the TPB and the evidence for increased explained variance when considering additional constructs such as habits, actual control and compensatory health beliefs, we expect these determinants to also be predictive of the consumption of prebiotic vegetables.

Thus far, information regarding the consumption of prebiotic vegetables at population level is scarce. For Belgium, the only data that are available have been collected via the Belgian Food Consumption Survey [16], which measured food consumption in a Belgian sample with two non-consecutive 24-h dietary recalls. According to this survey, the number of respondents consuming prebiotic vegetables on a recall day was very low (example: salsify: *n* = 12 (0.38%); Jerusalem artichoke: *n* = 8 (0.25%); artichokes: *n* = 10 (0.32%); and leek: *n* = 836 (26.57%), out of 3146 respondents) [17]. A pilot study on 472 Belgian students conducted by the authors of the current paper confirmed the low frequency of prebiotic vegetable consumption. This study found that the main predictors of consumption were intention, habits, past behaviour and actual control, while subjective norms and hedonic attitudes were significant predictors of intention. It was also noted that 13.8% of the respondents did not recognize the leek/artichoke category, and that 35.2% did not recognize the salsify/Jerusalem artichoke category. A student sample is however not representative of an entire population, and the prevalence and determinants of prebiotic vegetable consumption might be different for a representative sample because characteristics like age, gender and educational level are predictors of fruit and vegetable consumption in Belgium [2].

The current study aimed to document the prevalence of prebiotic vegetable consumption in a representative sample of the Walloon region in Belgium. Furthermore, it aimed to investigate if the consumption of prebiotic vegetables can be predicted by using an extended version of the Theory of Planned Behaviour including habits, actual control and compensatory health beliefs.

## Materials and methods

### Participants

An online questionnaire with an average duration of 20 min was administered in April/May 2016 to a representative sample of 1278 adults living in the Walloon region in Belgium, using a mixed approach (1078 online questionnaires and 200 face-to-face questionnaires with the help of a tablet). Face-to-face questionnaires were added to the online procedure to ensure the recruitment of older people and other people that do not use the Internet regularly. Representativeness of the Walloon region in Belgium was obtained in terms of gender, age crossed with gender, level of education crossed with gender, province, population density and (un) employment in 2016 based on information provided by the Walloon government by quota sampling. Table 1 summarizes the demographic characteristics of the sample compared to the entire population.

**Table 1:** Demographic characteristics of the representative sample of the Walloon region (Belgium) in the study and real-life

**Age crossed with sex**	Total	Women	Men
Age	100%	**52.2%**/52%	**47.8%**/48%
18–24	**11%/**11%	**10.6%**/10.6%	**10.6%**/11.7%
25–34	**16%**/16%	**15.9%**/15.1%	**16.2%**/16.5%
35–40	**10.1%**/10%	**9.9%**/9.6%	**10.3%**/10.4%
41–54	**24.2%**/25%	**23.1%**/23.7%	**25.4%/**25.5%
55–60	**10.1%/**10%	**9.7%/**9.7%	**10.5%/**9.9%
61 +	**28.6%/**29%	**30.7**%/31.4%	**26.2%/**26.0%
**Education level**	Total	Women	Men
Without diploma, primary school, lower secondary education	**41.6%**/42%	**42.6%**/44%	**40.6%/**41%
Higher secondary education	**28.9%**/29%	**27.9%**/27%	**30.0%/**30%
Higher education	**29.5%**/29%	**29.5%**/29%	**29.5%/**29%
**Urbanisation**	Total		
Rural (− 500 hab./km^2^)	**55.2%**/53%		
Suburban (500–1000 hab./km^2^)	**19.2%**/20%		
Urban (1000+ hab./km^2^)	**25.5%**/27%		40
**Province**	Total		
Brabant wallon	**11.0%**/11%		
Hainaut	**36.7%**/37.2%		
Liège	**32%**/30.5%		
Namur	**13.1%**/13.6%		
Luxembourg	**7.1%**/7.8%		
**Employment**	Total		
Employed	**75.8%**/76%		
Un-employed	**24.2%/**24%		

Note. Percentages for the participants in the study in bold, percentages for the population of Wallonia in 2016 in regular typeset

### Data collection

Recruitment of the sample and data collection was carried out by an independent market research/opinion poll company (Dedicated). The questionnaire was implemented online using Net Survey software (version 7). For the selection of the representative sample, a first mail was sent out to 14,000 people with different profiles in the company’s database. Of the persons who responded, 660 could not participate because the quota for their profile of demographic variables had already been reached. The 1108 responses of the final group of online participants were controlled for quality by looking at the duration of completion, incoherent responses, or whether participants systematically gave the same response. This resulted in the deletion of 30 records. The resulting sample of 1078 respondents was completed by selecting a sample of 200 participants, who were interviewed face-to-face. For these interviews, seven experienced interviewers went door-to-door and helped respondents complete the questionnaire on a tablet. When a person chose not to participate in the study, the interviewer went to the next house in the street. The response rate for the face-to-face interviews was 25%. At the end of the data collection, the interviewers targeted people with specific demographic profiles to ensure representativeness of the entire sample.

### Measures

The questionnaire for the study had been developed and validated on a pilot sample of 472 students. In addition to demographic characteristics (age, gender, BMI, nationality, highest degree or level of school completed, subjective income, main residence, and food restrictions and intolerances) it measured the consumption of vegetables in general and of prebiotic vegetables and its determinants, with leek representing the category prebiotic vegetables that were relatively often consumed and more familiar, and salsify representing the category that were less often consumed and less familiar. For each of the three categories (vegetables in general, leek, and salsify), the following questions were formulated.

### Outcome variables

Consumption frequency. Vegetable consumption was measured by a single item: “How often do you eat vegetables or salad (without counting vegetable juice or potatoes)? Think about your average consumption over the last 12 months”. The item was measured on a 7-point scale ranging from 1 (less than once per month) to 7 (more than once per day). Consumption frequency of prebiotic vegetables was measured by a single item each for leek and salsify: “How often do you eat the following vegetable? Think about your average consumption over the last 12 months”, measured on a 10-point scale ranging from 1 (never) to 10 (more than once per day).

Intention was measured by a single item for each category: “My intention to eat more vegetables/leek/salsify in the future is …” , measured on a 5-point Likert scale ranging from 1 (very weak) to 5 (very strong).

### Predictor variables

Rational attitudes were measured by two items for vegetables in general only (e.g., “Eating vegetables contributes to a longer life”, measured on a 5-point Likert scale ranging from 1 (don’t agree at all) to 5 (totally agree). The Cronbach’s alpha was .53.

Hedonic attitudes were measured by two items for vegetables, four items for leeks, and three items salsify (e.g., “Eating vegetables/leek/salsify is unpleasant”), using a 5-point Likert scale ranging from 1 (don’t agree at all) to 5 (totally agree). Cronbach alphas were .67 for vegetables, .92 for leek and .94 for salsify. The number of items varied for each category because exploratory factor analyses in the pilot study showed that certain items formed a more reliable scale that varied per category.

Subjective norms were measured with three items for vegetables and two items for leek and salsify, using a 5-point Likert scale ranging from 1 (don’t agree at all) to 5 (totally agree) (e.g., “My family thinks I should eat vegetables/leek/salsify”). Cronbach alphas were .93 for vegetables, .93 for leek and .94 for salsify.

Habits were measured by three items on a 5-point Likert scale ranging from 1 (don’t agree at all) to 5 (totally agree) for each category (e.g., “I eat vegetables/leek/salsify because they are a habit for me to eat”). Cronbach alphas were .80 for vegetables, .82 for leek and .90 for salsify. Respondents that indicated that they never eat leek or salsify were automatically assigned the lowest possible score.

Perceived control was measured by four items for vegetables and by six items for leek and salsify. (e.g., “I do not eat vegetables/leek/salsify because they are difficult to prepare.”) The items were measured on a 5-point Likert scale ranging from 1 (don’t agree at all) to 5 (totally agree). Cronbach alphas were .93 for leek, .93 for salsify and for .83 vegetables in general. The number of items varied for each category because exploratory factor analyses in the pilot study showed that certain items formed a more reliable scale that varied per category.

Compensatory health beliefs (CHB) were measured by four items referring to food in general, based on an existing questionnaire by Knäuper et al. [12] (e.g. “You can eat without many restrictions if you eat vegetables”). The items were measured on a 5-point Likert scale ranging from 1 (do not agree at all) to 5 (totally agree). The Cronbach’s α for this scale was .73.

Actual control was measured by a single item for each category (“It is not entirely my decision to (not) eat vegetables/leek/salsify, for instance: the decision is taken by my parents”), measured on a 5-point Likert scale ranging from 1 (do not agree at all) to 5 (totally agree).

Perceived environmental barriers were measured by two single items assessing availability (“I do not eat vegetables/leek/salsify because they are not available in stores near me” and price (“I eat vegetables/leek/salsify because they are cheap”), respectively. These items were scored on a 5-point Likert scale ranging from 1 (don’t agree at all) to 5 (totally agree).

### Descriptive variable

Stages of change were measured by a single item per category: “Do you have the intention to eat more vegetables//leek/salsify in the future?” , with 5 response options representing the five stages of change: 1 (“I don’t have the intention to eat more”), 2 (“I could eat more”), 3 (“I have the intention to eat more”), 4 (“I already started to eat more during the last six months”), 5 (“It has been more than six months since I started to eat more than before”).

### Analyses

Descriptive analyses were performed on the outcome variables of intention, consumption and stage of change, followed by hierarchic multiple regression analyses to investigate the determinants of intention to consume and self-reported consumption. All analyses were conducted in IBM SPSS, version 22.

## Results

### Consumption

On average, the participants reported consuming vegetables 5 times per week (*M* = 5.21 on a scale of 7, *SD* = 1.26). Leek was consumed one to three times per month (*M* = 5.11 on a scale of 10, *SD* = 1.78) and salsify less than once every 3 months (*M* = 2.80, *SD* = 2.04) on average. Three participants (0.23%) did not recognize leek and were therefore excluded from the analysis of the determinants of leek consumption; 137 (12.71%) did not recognize salsify and were excluded from the analysis of the determinants of salsify consumption.

### Behavioural intention

The intention to consume more in the future was moderate for vegetables (*M* = 3.32, *SD* = 1.01) and leek (*M* = 2.88, *SD* = 1.06), and weak for salsify (M = 2.20, SD = 1.08). In terms of the stages of behavioural change as identified in the Trans-Theoretical Model, 21, 33 and 50.6% of the participants were in the pre-contemplation stage regarding the consumption of vegetables in general, leek and salsify, respectively; 36.1, 33.5 and 27.3% were in the contemplation stage; 17.4, 18.4 and 14.8% in the preparation stage; 8.7, 5.8 and 3.1% in the action stage; and 16.8, 9.3 and 4.2% in the maintenance stage.

### Determinants of intention

#### Vegetables in general

A hierarchical multiple regression analysis with the dimensions of the Theory of Planned Behaviour (TPB) (hedonic attitudes, rational attitudes, subjective norm, and perceived control) as predictor variables revealed that these dimensions significantly predicted the intention to eat more vegetables but explained only a small part of the variance (F (4, 1273) = 49.59, *p* = .00, *R*^2^ = .14). All four TPB-dimensions contributed to the relationship (subjective norms (ß = .10, *p* = .00), perceived control (ß = .08, *p* = .01), hedonic attitudes (ß = .21, *p* = .00) and rational attitudes (ß = .19, *p* = .00)). The addition of compensatory health beliefs as a predictor did not significantly increase the explained variance (Table 2).

**Table 2:** Determinants of intention to consume leek, salsify and vegetables in general

	Vegetables in general		Leek		Salsify	
	*ß*	*∆ R*^*2*^	*ß*	*∆ R*^*2*^	*ß*	*∆ R*^*2*^
Predictors						
**Step 1**		.14***		.16***		.26***
Hedonic Attitude	.21***		.38***		.44***	
Rational attitude	.19***		–		–	
Subjective norms	.10***		.23***		.31***	
Perceived control	.09*		.00		−.01	
**Step 2**		.00		.00		.00
CHB						

Note. *p < .05, ***p < .001, Leek: *N* = 1274, Salsify: *N* = 1140, Vegetables: *N* = 1277

#### Leek

A hierarchical regression analysis with the TPB-dimensions of hedonic attitudes, subjective norm, and perceived control as predictor variables revealed that these dimensions predicted the intention to consume leek but explained only a small part of the variance (F (3, 1271) = 78.03, *p* = .00, *R*^2^ = .16) with subjective norms (ß = .23, *p* = .00) and hedonic attitudes (ß = .38, *p* = .00) as significant predictors. Perceived control was not a significant predictor, (ß = .00, *p* = .99). Again, the addition of compensatory health beliefs as a predictor in a second step did not significantly increase the explained variance (Table 2).

#### Salsify

A hierarchical regression analysis with the TPB-dimensions hedonic attitudes, subjective norm, and perceived control as predictor variables revealed that these dimensions predicted the intention to consume salsify with a moderate amount of explained variance (F (3, 1137) = 134.72, *p* = .00, *R*^2^ = .26) with again subjective norms (ß = .31, *p* = .00) and hedonic attitudes (ß = .44, *p* = .00) as significant predictors. Perceived control was not a significant predictor, (ß = −.01, *p* = .81). Again, the addition of compensatory health beliefs as a predictor in a second step did not significantly increase the explained variance (Table 2).

### Determinants of consumption

#### Vegetables in general

A hierarchical multiple regression analysis with the demographic variables that were significant in a preliminary analysis (gender and age) as predictors of the consumption of vegetables in general produced a small but significant effect in terms of explained variance (F (2, 1268) = 51.11, *p* = .00, *R*^2^ = .08), with being older (ß = .22, *p* = .00) and female (ß = .16, *p* = .00) as significant predictors. The addition of the TPB-dimensions intention and perceived control as predictor variables increased the explained variance significantly (F change (2, 1266) = 118.26, *p* = .00, ∆ *R*^2^ = .15), with perceived control (ß = .35, *p* = .00), intentions (ß = .14, *p* = .00), being older (ß = .11, *p* = .00) and being female (ß = .07, *p* = .01) as significant predictors. In a next step, the addition of habits as a predictor further increased the explained variance significantly (F change (1, 1265) = 72.86, *p* = .00, ∆ *R*^2^ = .04), with perceived control (ß = .28, *p* = .00), habits (ß = .23, *p* = .00), intentions ß = .12, *p* = .00), being older (ß = .08, *p* = .00) and being female (ß = .07, *p* = .01) as significant predictors. Finally the effect of consumption increased slightly with the addition of availability, price and actual control (F change (3, 1262) = 2.79, *p* = .04, ∆ *R*^2^ = .01) with actual control (ß = .06, *p* = .04), perceived control (ß = .29, *p* = .00), habits (ß = .24, *p* = .00), intentions ß = .12, *p* = .00), being older (ß = .09, *p* = .00) and being female (ß = .07, *p* = .01) being significant predictors (Table 3). Price (ß = −.02, *p* = .60) and availability (ß = −.05, *p* = .06) were not significant predictors. The R^2^ of the final model was .28, which can be considered as a moderate amount of explained variance.

**Table 3:** Determinants of consumption of leek, salsify and vegetables in general in final model

	Vegetables in general (V)		Leek (L)		Salsify (S)	
	*ß*	*∆ R*^*2*^	*ß*	*∆ R*^*2*^	*ß*	*∆ R*^*2*^
Predictors						
**Step 1**		.08***		.04***		.02***
Age (V, L)	.09**		.05		–	
Gender (V, S)	.07**		–		−.07**	
Being vegan (L)	–		.06*		–	
Being vegetarian (L)	–		.02		–	
Diploma (L)	–		−.03		–	
Subject. income (S)	–		–		.02	
Province (Liege) (L)	**–**		.00		–	
**Step 2**		.15***		.15***		.17***
Perceived control	.29***		.11*		−.04	
Intention	.12***		.25***		.13***	
**Step 3**		.04***		.10***		.23***
Habits	.24***		.35***		.56***	
**Step 4**		.01*		.01**		.004*
Price	−.03		.04		.10**	
Actual control	.06*		.02		−.01	
Availability	−.05		.10**		−.06	

Note. *p < .05, **p < .01, ***p < .001, Leek: *N* = 1267, Salsify: *N* = 1133, Vegetables: *N* = 1270, *V* vegetables in general, *L* leek, *S* salsify

#### Leek

A hierarchical multiple regression analysis with the demographic variables that were significant in a preliminary analysis (province in Belgium (Liege), food restrictions (being vegetarian and vegan), diploma, age) as predictors of the consumption of leek produced a small but significant effect in terms of explained variance (F (5, 1262) = 9.34, *p* = .00, *R*^2^ = .04), with being vegan (ß = .07, *p* = .02) and older age (ß = .17, *p* = .00) as significant predictors. The strength of this relationship significantly increased with the addition of intentions and perceived control (F change (2, 1260) = 114.04, *p* = .00, ∆ *R*^2^ = .15) with intention (ß = .38, *p* = .00), being vegan (ß = .07, *p* = .01) and older age (ß = .10, *p* = .00) as significant predictors. The strength of this relationship significantly increased with the addition of habits (F change (1, 1259) = 179.42, *p* = .00, ∆ *R*^2^ = .10), with habits (ß = .36, *p* = .00), intention (ß = .25, *p* = .00), and being vegan (ß = .05, *p* = .03) as significant predictors. The amount of variance explained increased slightly with the addition of availability, price and actual control (F change (3, 1256) = 4.20, *p* = .01, ∆ *R*^2^ = .01), with availability (ß = .10, *p* = .01), perceived control (ß = .11, *p* = .01), habits (ß = .35, *p* = .00), intention (ß = .25, *p* = .00) and being vegan (ß = .06, *p* = .02) as significant predictors of leek consumption (Table 3). Living in Liege (ß = .00, *p* = .90), being vegetarian (ß = .02, *p* = .35), age (ß = .05, *p* = .07), diploma (ß = −.03, *p* = .18), actual control (ß = .02, *p* = .54) and price (ß = .04, *p* = .33) were not significant predictors. The R^2^ of the final model was .30, which can be considered as a moderate amount of explained variance.

#### Salsify

A hierarchical multiple regression analysis with the demographic variables that were significant in a preliminary analysis (subjective income and gender) as predictors of the consumption of salsify produced a small but significant effect in terms of explained variance (F (2,1131) = 10.67, *p* = .00, *R*^2^ = .02), with the male gender (ß = −.12, *p* = .00) being a significant predictor. This strength of this relationship significantly increased with the addition of intention and perceived control (F change (2, 1129) = 115.80, *p* = .00, ∆ *R*^2^ = .17), with intention (ß = .41, *p* = .00) and being male (ß = −.11, *p* = .00) as significant predictors. This effect size further increased substantially with the addition of habits (F change (1, 1128) = 432.37, *p* = .00, ∆ *R*^2^ = .23), with habits (ß = .56, *p* = .00), perceived control (ß = −.08, *p* = .00), intention (ß = .14, *p* = .00), and being male (ß = −.07, *p* = .00) as significant predictors. Finally, the effect size increased slightly with the addition of availability, price and actual control (F change (3, 1125) = 2.84, *p* = .04, ∆ *R*^2^ = .004) with price (ß = .10, *p* = .01), habits (ß = .56, *p* = .00), intention (ß = .13, *p* = .00) and being male (ß = −.07, *p* = .00) as significant predictors (Table 3). Subjective income (ß = .02, *p* = .34), perceived control (ß = −.04, *p* = .33), actual control (ß = −.01, *p* = .84) and availability (ß = −.06, *p* = .12) were not significant predictors. The R^2^ of the final model was .42, which can be considered as a moderate amount of explained variance.

## Discussion

The aim of the study was to document the prevalence and determinants of prebiotic vegetable consumption in a representative sample of Wallonia, compared to those of vegetables in general.

### Descriptives

The findings of our study confirm those of other studies ([17], pilot study) that salsify is not well known as a vegetable amongst the Walloon population in Belgium, to the extent that more than one out of ten persons (12.71%) do not recognize them when presented with their name and picture. It is not surprising, then, that salsify is not often consumed (less than once every 3 months), and that there is only a weak intention to consume more salsify in the future, with half the respondents in this sample (50.6%) not even contemplating (stage 1) to eat more salsify. Leek, on the other hand, is a more familiar vegetable, with only 0.23% of the participants in our sample not recognizing it. Nevertheless, it remains seldom consumed (one to three times per month), and the intention to consume more remains only moderate. This is in contrast with vegetables in general, which are consumed more often (five to six times per week), although the level of consumption does not meet the recommendation of the WHO in terms of frequency of daily consumption of fruits and vegetables [3]. While there is only a moderate intention to consume more vegetables, a substantial proportion of the participants (33.5 and 36.1%, respectively) were in the contemplation stage for both leek and vegetables in general. It is clear that for all vegetable categories an increase in both intentions and actual behaviour is necessary to experience the health benefits of (prebiotic) vegetables.

### Demographical predictors

Being female and older are predictors of vegetable consumption, in line with the results of the Belgian Health Interview Survey conducted in 2018 [2]. Furthermore, being vegan predicts leek consumption. This is not surprising, as vegans probably eat a wider range of vegetables to increase variety in their meals. More surprisingly, being male was found to predict a higher consumption of salsify.

### Socio-psychological determinants

In addition to the aforementioned demographic factors, the intention to consume prebiotic vegetables is also influenced by two socio-psychological factors, namely hedonic attitudes and subjective norms. For vegetables in general, but not for the prebiotic vegetables, perceived control and rational attitudes are also significant determinants. Rational attitudes were not measured for the prebiotic vegetables, however, because internal reliability of the scale was too low for these vegetables so it is not possible to compare vegetables in general to prebiotic vegetables on this dimension. The finding that hedonic attitude, which in our study mainly referred to taste and texture, is the most important predictor of intention across all vegetable categories is in line with previous work on vegetables in general [12, 13]. The non-significant role of perceived control as a predictor for the intention to consume prebiotic vegetables, on the other hand, is interesting, especially since the mean score for perceived control was high for all categories. This suggests that although people experience a subjective feeling of control over prebiotic vegetable consumption, the intention to actually consume them depends more on the expected pleasure and taste, and on beliefs about what others think about these vegetables. Furthermore, we note that compensatory health beliefs (CHB) did not contribute to the prediction of the intention to consume vegetables of any category. However, as the participants in our study had a low average score and standard deviation on CHB, it is possible that the lack of a significant effect was due to the low variability of the scores in our sample. As such, there is a need for more research of this topic using a more sensitive measure. The explained variances of the final models were small (vegetables: *R*^2^ = .14, leek: *R*^2^ = .16) to moderate (salsify: *R*^2^ = .26) showing that the TPB constructs seem to be more important predictors for the unfamiliar prebiotic vegetable in comparison to the familiar (prebiotic) vegetable.

With regard to actual consumption, habits and intentions were significant predictors of consumption for all three categories. However, in each case, intention was a weaker predictor than habit. This confirms a previous meta-analysis [18] that showed that habits are the most important moderators of the intention-behaviour relationship. When circumstances did not support habit formation, intentions had a strong effect on behaviour (d = .74). However, when circumstances supported the development of habits this effect size dropped substantially (d = 0.22) [18]. Especially for the unfamiliar prebiotic vegetable salsify, it seems that habits are a strong predictor of consumption in the final model that greatly increased the explained variance.

Perceived control was a significant predictor of the consumption of leek and vegetables in general, but not for salsify. For the latter, contextual factors such as a perceived high price seem to be more relevant, in addition to habits and intentions. The wide availability of leeks is a predictive factor of leek consumption, while actual control (i.e., the extent to which one decides what to eat instead of others) predicts only vegetable consumption in general. In sum, whereas both motivational factors like intention and habitual responses are important determinants of the consumption of all vegetables, contextual factors such as price, availability and actual control have a more specific influence on certain prebiotic vegetables. The final models of all categories were considered to explain a moderate amount of explained variance for all categories (vegetables: *R*^2^ = .28, leek: *R*^2^ = .30, salsify: *R*^2^ = .42) but it is again the consumption of the unfamiliar prebiotic vegetable salsify that was best explained by the TPB constructs, habits and perceived environmental barriers.

Based on the findings from this study, there are several determinants that could be targeted in interventions to increase the consumption of prebiotic vegetables. It seems that for both leek and salsify, intentions are a predictor of behaviour. Hedonic attitudes and subjective norms are important targets to influence these intentions. Hedonic attitudes concerning taste and texture could be increased by offering tasty samples of leek and salsify, or integrating them into dishes. For instance, one study that offered participants a prebiotic diet for 2 weeks, showed a trend toward an increased hedonic attitude regarding salsify consumption after the diet [19]. Subjective norms are more difficult to influence for family and friends, but general norms can be influenced by techniques such as social modelling in the media. In this case, models would demonstrate a recommended behaviour and the assumption is that people will start engaging in this behaviour when they observe other people performing this behaviour [20]. Another target for behaviour change could be aimed at forming a habit, as habit seems to be a strong predictor for both salsify and leek consumption. Habits develop as people repeatedly perform a specific behaviour in a stable situation to pursue their goals [15]. The formation of habitual prebiotic vegetable consumption could for instance be aided with the use of implementation intentions. Implementation intentions are simple action plans stipulating where, when, and how one will perform an intended behaviour [21]. Finally, changing contextual factors in a positive way, by increasing the availability or decreasing price, could also increase prebiotic vegetable consumption.

### Strengths and limitations

This study is, to our knowledge, the first to investigate the determinants of prebiotic vegetable consumption drawing on psychological models. However, it is not without limitations. A first limitation is that it is correlational, with intention and behaviour measured at the same point in time for practical reasons. This precluded making inferences regarding causal relationships. However, to investigate relatively unknown behaviour such as specific vegetables, correlational research is an important first step before embarking on intervention studies [22]. While prospective designs are believed to allow for a stronger test of the Theory of Planned Behaviour, this is not the case if the causal lag between predictors and outcomes is very short [23]. As the TPB model assumes a very short causal lag [23], a correlational design is appropriate. Another limitation is that the behaviour is self-reported. It is well known that people are not very accurate in assessing their consumption of food over a long time period. However, prebiotic vegetables are very specific and possibly seasonally consumed, therefore asking for a recall of their consumption over a long time period seems warranted. As psychological research is usually performed in a convenience (student) sample, the representativeness of the current study is an important strength. At the same time, this makes the results less generalizable to other countries. It is possible that results are generalizable to similar countries in terms of representativeness (gender, age, level of education, population density and (un)employment), but other aspects such as culture and gross domestic product should also be considered.

## Conclusions

For future studies it is advisable to take into account that determinants for consumption may not be the same for all “fruit and vegetables” or “vegetables”. These umbrella terms undermine the differences between the specific foods regarding their demographical and socio-psychological determinants. By implementing interventions aimed at the determinants of general categories of vegetables, some specific vegetable consumption might remain unchanged. For instance, the influence of perceived control and rational attitudes is stronger for vegetables in general than for specific, prebiotic vegetables. The influence of specific environmental factors such as price, availability and actual control also differ depending on the type of vegetable. The findings of this study can be used as a target in interventions to increase (prebiotic) vegetable consumption.

## Data Availability

The datasets used and/or analysed during the current study are available from the corresponding author on reasonable request.

## Abbreviations

CHB: Compensatory health beliefs
ITF: Inulin-type fructan
TPB: Theory of Planned Behaviour
TTM: Trans-Theoretical Model of change
